# Clinical Utility of TRBC1 Addition in Multiparametric Flow Cytometry for T Cell Lymphoma Diagnosis

**DOI:** 10.37825/2239-9747.1059

**Published:** 2024-08-16

**Authors:** Francesca Picone, Marisa Gorrese, Angela Bertolini, Bianca Serio, Luca Pezzullo, Roberto Guariglia, Pio Zeppa, Alessandro Caputo, Albino Carrizzo, Carmine Vecchione, Carmine Selleri, Valentina Giudice

**Affiliations:** a Hematology and Transplant Center, University Hospital “San Giovanni di Dio e Ruggi d’Aragona”, 84131 Salerno, Italy; b Department of Medicine, Surgery, and Dentistry, University of Salerno, 84081 Baronissi, Italy; c Anatomy Pathology Unit, University Hospital “San Giovanni di Dio e Ruggi d’Aragona”, 84131 Salerno, Italy; d Vascular Physiopathology Unit, IRCCS Neuromed, Pozzilli (IS), Italy

**Keywords:** T cell lymphoma, TCR clonality, Flow cytometry

## Abstract

Diagnosis of T cell lymphoproliferative disorders requires one or multiple immunophenotypic aberrations, histological tissue structure or composition modifications, and T cell clonality demonstration. Here, we present two T cell lymphoma cases, where TCR clonality was evaluated using the TRBC1 monoclonal antibody combined with a multi-parametric staining for an in-depth immunophenotype of physiological and pathological T cell populations. In the first case, this monoclonal antibody allowed exclusion of reactive lymphoproliferations, while in the second case, it was conclusive for identification of Sezary syndrome cells. We added evidence on clinical utility of TRBC1 antibody (JOVI-1) to disclose monotypic T-cell populations, and TCR clonality evaluated by TRBC1 monoclonal antibody can be efficiently combined with a multi-parametric staining for an in-depth immunophenotype, with high versability of this monoclonal antibody in multi-parametric flow cytometry panel.

T-cell neoplasms are a heterogeneous uncommon and rare subtype of non-Hodgkin lymphoproliferative disorders with different clinical presentations, ranging from indolent to vary aggressive entities, phenotypic and histological features, and involved tissues or organs, according to the 2022 World Health Organization classification [1]. Primary T-cell lymphomas can affect lymph nodes, skin, or extramedullary sites, such as intestine, liver, or oral cavity, while peripheral blood and bone marrow are more commonly secondarily involved. However, differential diagnosis between T cell neoplasms is still challenging, especially with reactive lymphoproliferation, because of the lack of subtype-specific immunophenotyping or immunohistochemistry markers of neoplastic T cell clones [1]. Normal T lymphocytes are characterized by surface expression of CD2, CD7, CD3, and CD5, and mature T cells by an αβ-(95% of total peripheral blood circulating cells) or γδ-chains (5% of total peripheral blood circulating cells) T-cell receptor (TCR) rearrangement. In physiological conditions, TCR repertoire is polyclonal, as each T cell carries a TCR with a specific rearrangement with different antigen affinity, allowing the recognition of more than 10^18^ known and unknown epitopes [2]. β chain assembling derives from a complex gene locus rearrangement, known as VDJ recombination, obtained froma random rearrangement of one Vβ locus among 52 available with one Dβ (between Dβ1 or 2), one Jβ (among 6 Jβ1 and 7 Jβ2 loci), and one constant Cβ region (between Cβ1 or 2) [2,3]. On a rearranged VDJ, a TdT (terminal deoxynucleotidyl transferase) enzyme produces insertions/deletions by adding or removing nucleotides within a hypervariable region termed complementary determining region 3 (CDR3), between V-D and D-J regions, thus increasing TCR variability and antigen-recognition ability [3,4]. Therefore, because of this VDJ region highly variability, in physiological conditions and in early-stage infections, TCR repertoire is polyclonal; conversely, in reactive conditions during immunological tumor-specific responses, TCR repertoire is oligoclonal, because of the presence of few expanded immunodominant clones carrying a TCR with high tumor antigen-affinity. Indeed, TCR clonality assessment is an essential diagnostic tool for certain T-cell lymphomas, such as T-large granular lymphocytic leukemia, while its utility in differential diagnosis of T cell neoplasms is still under investigation, especially in those clinical entities without specific flow cytometric neoplastic markers [1]. TCR repertoire analysis can be performed at the DNA level, covering the entire TCR sequence depending on probes and restriction enzyme used; however, TCR repertoire analysis at the protein level by flow cytometry covers less than 70% of all variable region domains grouped in 24 families and uses eight pre-mixed antibody staining (e.g., Beta Mark TCR Vbeta Repertoire Kit, Beckman Coulter) [2]. Conversely, the recently described JOVI-1 clonemonoclonal antibody is highly specific to the TCR β-chain constant region 1 (TRBC1) domain, and allows TCR repertoire clonality assessment using a single fluorochrome-conjugated antibody in combination with other surface markers for a deeper immunophenotypic definition of studied populations [1]. However, its use in routinely clinical practice is still under investigation, despite recent evidence of its utility in differential diagnosis of T cell lymphomas [5]. Here, we added evidence of clinical utility of TRBC1 monoclonal antibody in multi-parametric flow cytometry for TCR clonality assessment in normal and pathological populations and for assessing diagnosis of T cell neoplasms.

The first case was a 68-years old male who arrived at our observation in September 2023 for axillary and inguinal lymphadenopathy. A fine-needle aspiration cytology (FNAC) was preliminary performed for excluding reactive lymphoproliferations, and immunophenotyping was also carried out on 100 μL of specimen with the following antibodies according to manufacturers, instructions: TRBC1 (FITC), CD1a (PE), CD2 (APC), CD3 (ECD), CD4 (FITC, PE, or APC), CD7 (PC7 or APC700), CD8 (APC700 or APC750), CD10 (PC7), CD16 (Pacific Blue), CD19 (PC5.5 or Pacific Blue), CD25 (PC5.5), CD26 (PE), CD38 (PC7), CD45 (PC5 or Krome Orange), CD45RA (PC5.5), CD49d (FITC), CD56 (ECD), CD57 (FITC), CD123 (PE), CD200 (PC7), Programmed cell death protein 1 (PD-1) (PE), PD-L1 (PC7), TCRαβ (PE), TCRγδ (FITC), HLA-DR (FITC), CCR7 (FITC), and TdT (FITC). After incubation, red cell lysis was performed with IO Test Lysing Solution (Beckman Coulter), cells were washed twice with phosphate buffer saline (PBS, IsoFlow Sheath Fluid, Beckman Coulter), and then resuspended in 500 μL of the same buffer for acquisition. Samples were acquired on a NaviosEX cytometer (Beckman Coulter), equipped with violet (405 nm), blue (488 nm), and red (638 nm) lasers. Instrument daily quality control was performed using Flow-Check Pro Fluorospheres (Beckman Coulter), and external quality control by UK NEQAS for Leucocyte Immunophenotyping. Compensation was monthly checked as per clinical laboratory practice using flow-set and compensation kit (Beckman Coulter). Compensation was calculated using single-color controls for each fluorochrome, and an unstained sample was used as negative control for setting PMT voltages. Samples were run using the same PMT voltages, and at least 1,000,000 events were recorded. Post-acquisition analysis was carried out using Kaluza C 1.1 software (Beckman Coulter). For neoplastic cell identification, double cells were first removed using linear parameters and time, and then by forward scatter area (FSC-A) and CD45 expression. In this first case, neoplastic cells from FNAC specimen were FSC^high^ SSC^high^ CD45^+^ CD3^+^ CD7^−^, and also showed positivity for TCRαβ (99%), CD4 (99%), CD10 (1%), CD49d (99%), CD200 (63%), and PD1 (99%), while negativity for TCRγδ (1%), CD26 (1%), TdT (1%), CD57 (1%), CD56 (1%), CD38 (14%), PD-L1 (1%), and for TRBC1 (1%) by flow cytometry (Fig. 1A). Our multi-parametric panel was designed to exclude the presence of Natural Killer (NK) cells (positivity for CD57, CD56, and CD16, and negativity for TRBC1) and to simultaneously assess TCR clonality on both normal CD7^+^ and pathological CD7^−^ cells. Normal T cell counterpart was polyclonal for TRBC1, while pathological cells were negative. Indeed, polyclonal expression of TRBC1 is improbably found on aberrant T cells in peripheral T-cell lymphomas, thus helping in differential diagnosis [5,6]. CD200, a type 1 transmembrane glycoprotein physiologically expressed on a variety of tissues (e.g., nerves, vascular endothelium, B cells, or follicular dendritic cells) was included in our panel, because its aberrant expression on T cells is frequently associated with angioimmunoblastic T cell lymphoma, T cell acute lymphoblastic leukemia, and early T cell precursor acute lymphoblastic leukemia [6,7]. PD-1 is currently routinely used for diagnosis of peripheral T-cell lymphomas. CD49d, also known as very late antigen-4 or α4β1, was also studied, as this is a marker of adhesion, migration, and homing, and associated with poor prognosis when is aberrantly expressed on neoplastic clones [8,9]. In normal individuals, circulating lymphocytes maintain this integrin in a low-affinity state, unable to interact with its ligands. Its absence is an exclusive feature on circulating Sézary syndrome cells, suggesting an impairment of peripheral blood T cells to correctly migrate to the skin. However, its expression on other T cell lymphomas obtained froma FNAC specimen has not been described yet, as well as its possible pathogenic role [10–12]. In angioimmunoblastic lymphoma, PD-1 shows a bright expression and can help in differential diagnosis with other peripheral T-cell lymphomas, as well as inclusion of CD10 in multi-parametric flow cytometry panels allowing differential diagnosis of angioimmunoblastic lymphoma because of its lack, and CD200 inclusion for ruling out a diagnosis of nodal T-follicular helper cell lymphoma [13,14]. Based on flow cytometry immunophenotyping of FNAC and histological findings of lymph node biopsy that confirmed the presence of a CD3^+^CD7^−^ pathological T-cell population, and bone marrow specimens negative for lymphoid infiltration, the patient was diagnosed with a stage IIIA T-cell lymphoma not-otherwise, based on current diagnostic immunophenotypic criteria: CD4>CD8; frequent antigen loss (CD5 or CD7); CD30^+/−^ and CD56^+/−^; subset follicular T helper features; and cytotoxic granules^+/−^ [15]. In other studies, TRBC1 has been added to custom panels including CD2/CD3/CD4/CD5/CD7/CD8/CD16/CD26/CD45/TRBC1 or CD2/CD3/CD4/CD5/CD7/CD8/CD45/TCRγδ/TRBC1 [16–18]. In our case, inclusion of TRBC1 in a multiparametric flow cytometric panel consisting of TRBC1/PD-1/CD3/CD7/CD4/CD8/CD45 helped in neoplastic clone immunophenotyping together with additional panels for CD10 and CD200 expression analysis for differential diagnosis between peripheral T-cell and angioimmunoblastic lymphoma. Indeed, this panel allowed a rapid confirmation of the presence of a neoplastic CD3^+^CD7^−^CD4^+^ clone with bright PD-1 expression and absence of TRBC1, and a simultaneous comparison of TRBC1 restriction in normal CD3^+^CD7^+^CD4^+^ counterpart.

The second case was a 64-years old female with a diagnosis of Sézary syndrome received in 2006 who was first treated with interferon and photopheresis, with alemtuzumab followed by gemcitabine in 2007, and then with 16 cycles of pentostatin in 2008 with clinical remission of the erythematous cutaneous rash and pruritus until August 2021. She arrived at our observation in February 2023 with a diffuse erythematous rash covering >70% of body surface and pruritus, and a peripheral blood immunophenotyping was performed to confirm the previous diagnosis of Sézary syndrome. Briefly, 50 μL of sample were stained with the following antibodies according to manufacturers, instructions: TRBC1 (FITC), CD2 (APC), CD3 (FITC or ECD), CD4 (FITC, PE, or APC), CD5 (PC7 or APC750), CD7 (PC7), CD8 (PC5 or APC700), CD16 (PB), CD19 (PC5.5), CD26 (PE), CD45 (ECD or Krome Orange), CD56 (ECD), CD123 (PE), CD200 (PC7), and PD-1 (PE). In the second case, peripheral blood neoplastic cells were SSC^low^CD3^dim^CD4^+^ with an unusual CD7^high^ expression for a Sézary syndrome, thus this case was a diagnostic challenge (Fig. 1B). Currently, Sézary cell identification by flow cytometry requires at least one WHO-EORTC 2018 classification for primary cutaneous lymphomas criteria among: (i) ≥1000 Sézary cells/μL in peripheral blood; (ii) a CD4/CD8 ratio ≥10; (iii) neoplastic CD4^+^CD7^−^ cells ≥30% or CD4^+^CD26^−^ cells ≥40%; and (iv) aberrant phenotype with loss or one or more T-cell antigens. A recent study has also proposed the inclusion of TRBC1 in a panel for T-cell lymphoma screening, including CD45, CD4, CD2, CD5, TCRγδ, CD7, CD3 and TRBC1 [15]. However, in our case, this multi-parametric panel would have not been sufficient to correctly identify neoplastic cells. Indeed, under reactive/non-neoplastic conditions, CD26 and CD7 loss of expression can be observed in CD4^+^ T cells. In our patient, CD26 was negative while CD7 was highly expressed; therefore, additional markers were investigated for differential diagnosis, including CD200 (negative), T cell-associated CD2 and CD5 (both expressed), NK markers CD16 and CD56 (both negative), a tumor-associated coinhibitory molecule PD-1 (highly expressed) with negativity of its ligand PD-L1, and TRBC1 for T cell clonality assessment. In contrast with the previous case, TRBC1 was positive, as confirmed by polyclonality of normal cells. Moreover, this patient showed CD200 negativity, that might occur in ~40% of Sézary syndrome subjects [15]. Therefore, in these patients, an additional marker for differential diagnosis might be useful, such as TRBC1 as in our case.

Diagnosis of T cell lymphoproliferative disorders requires one or multiple immunophenotypic aberrations, histological tissue structure or composition modifications, and T cell clonality demonstration [5,19]. In these two case reports, we added evidence on clinical utility of TRBC1 antibody (JOVI-1) to disclose monotypic T-cell populations, where TCR clonality was evaluated using the TRBC1 monoclonal antibody combined with a multi-parametric staining for an in-depth immunophenotype of physiological and pathological T cell populations. In the first case, this monoclonal antibody was studied in a FNAC sample together with other T cell neoplasm-associated markers to exclude reactive lymphoproliferations. In the second case, this monoclonal antibody was used for identification of atypical Sézary syndrome cells. Future directions include evaluation of both TRBC1 and TRBC2 to improve sensitivity and specificity of T cell clonality assessment in routine clinical practice.

## Figures and Tables

**Fig. 1:**
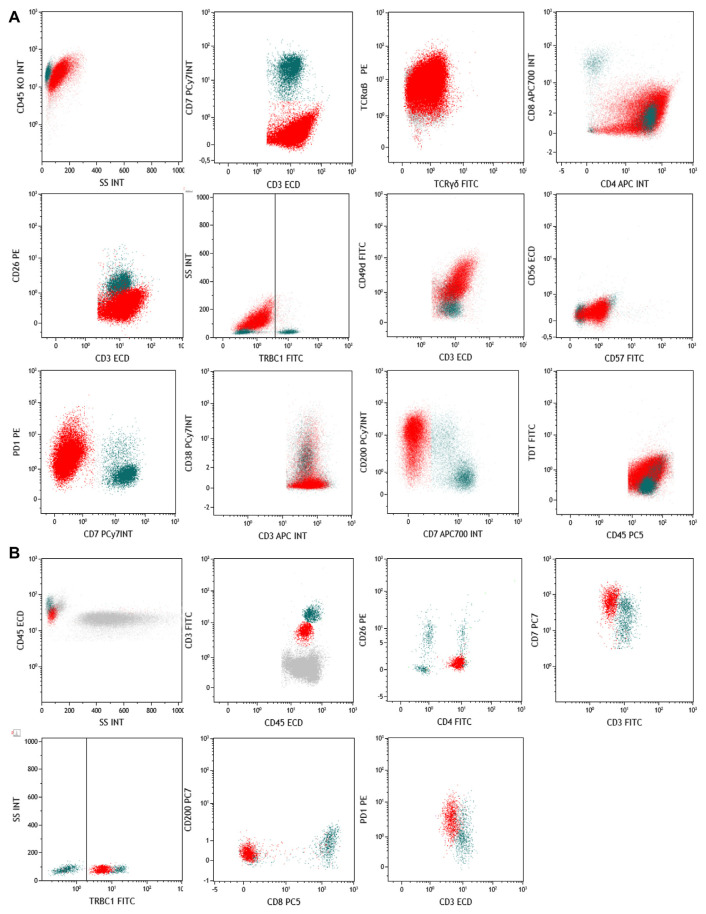
(A) Gating strategy of the fine needle aspiration cytology specimen obtained from inguinal lymph node. T cells were gated for CD7 and CD3 expression, and CD7^−^CD3^+^ pathological cells (in red) were further characterized for TCRαβ, TCRγδ, CD8, CD4, CD26, CD49d, CD56, CD57, PD-1, CD38, CD200, and TdT expression. Non-malignant counterpart is displayed in green. Furthermore, cells were studied for T cell receptor (TCR) clonality using a side scatter area (SSC) vs TRBC1 gating strategy. Pathological cells showed TCR negative restriction, while normal lymphocytes were polyclonal. As TRBC1^−^ population for setting gate boundary, normal lymphocyte counterpart was used, while normal lymphocytes (in green) were considered for setting the threshold to define TRBC1 negativity and positivity. (B) Gating strategy of the peripheral blood specimen from Sézary syndrome. Neoplastic cells were gated for CD3 and CD45 expression, and CD3^dim^ cells (in red) were further characterized for CD26, CD4, CD7, CD200, CD8 and PD-1 expression. Circulating Sezary CD7^+^CD26^−^ cells also showed TCR positive restriction, while normal counterpart (in green) was polyclonal.

## Data Availability

Data are available upon request by the authors.
